# Supplementary material for: Charge density waves tuned by biaxial tensile stress
Source: Nat Commun. 2024 Apr 30;15:3667. doi: 10.1038/s41467-024-47626-5 (PMC11063040; doi:10.1038/s41467-024-47626-5)
Supplement: Supplementary file 1 — Supplementary Information [file 41467_2024_47626_MOESM1_ESM.pdf]

Supplementary Information
Charge Density Waves Tuned by Biaxial Tensile Stress

A. Gallo-Frantz, V.L.R. Jacques*,* A.A. Sinchenko, D. Ghoneim, L. Ortega, and D. Le Bolloc'h
Laboratoire de Physique des Solides, Université Paris-Saclay, CNRS, 91405 Orsay Cedex, France

P. Godard and P.-O. Renault
Institut Pprime, CNRS-Université de Poitiers-ENSMA, 86962 Futuroscope-Chasseneuil Cedex, France

P.D. Grigoriev
L. D. Landau Institute for Theoretical Physics, Chernogolovka, Moscow Region 142432, Russia and National University of Science and Technology "MISiS", 119049, Moscow, Russia

A. Hadj-Azzem, J. E. Lorenzo, and P. Monceau
Univ. Grenoble Alpes, CNRS, Grenoble INP, Institut Néel, 38000 Grenoble, France

D. Thiaudière
Synchrotron SOLEIL, L'Orme des Merisiers, 91190 Saint-Aubin, France

E. Bellec
CEA Grenoble, IRIG, MEM, NRS, 17 rue des Martyrs, F-38000 Grenoble, France

CONTENTS

I. Supplementary Methods 2
A. Cryogenic Biaxial Tensile Stress Device 2
1. Description of the device 2
2. Characterization of strain in uniaxial and equibiaxial modes 3
B. Analysis of XRD data 5
1. Determination of lattice parameters from XRD data 5
2. Analysis of CDW peaks 9
C. Transport measurements 10
1. Resistance measurement in the Montgomery configuration 10
2. Conversion of resistances into resistivities 11
3. Determination of resistivity jumps 13
4. Determination of T_c 14
D. Additional results - Sample 2 16
E. Temperature dependence of electron Susceptibility 19

II. Supplementary References 19

References 19

* vincent.jacques@universite-paris-saclay.fr

I. SUPPLEMENTARY METHODS

A. Cryogenic Biaxial Tensile Stress Device

In this section, we provide more details about the Cryogenic Biaxial Tensile Stress (CryoBiaS) device developed at Laboratoire de Physique des Solides, and used for the experimental work shown in this paper.

1. Description of the device

The CryoBiaS system has been designed to fit a Konti Micro cryostat from CryoVac GmbH which has a cylindrical cold finger that keeps the sample at the same radial position even when thermal contraction/expansion takes place when changing the temperature. The cross-shaped CryoBiaS housing is made to be vacuum-tight when used with its cover, with usual working pressures $\sim 1 \cdot 10^{-6}$ mbar, compatible with the cryogenic environment (see Suppl. Fig. 1).

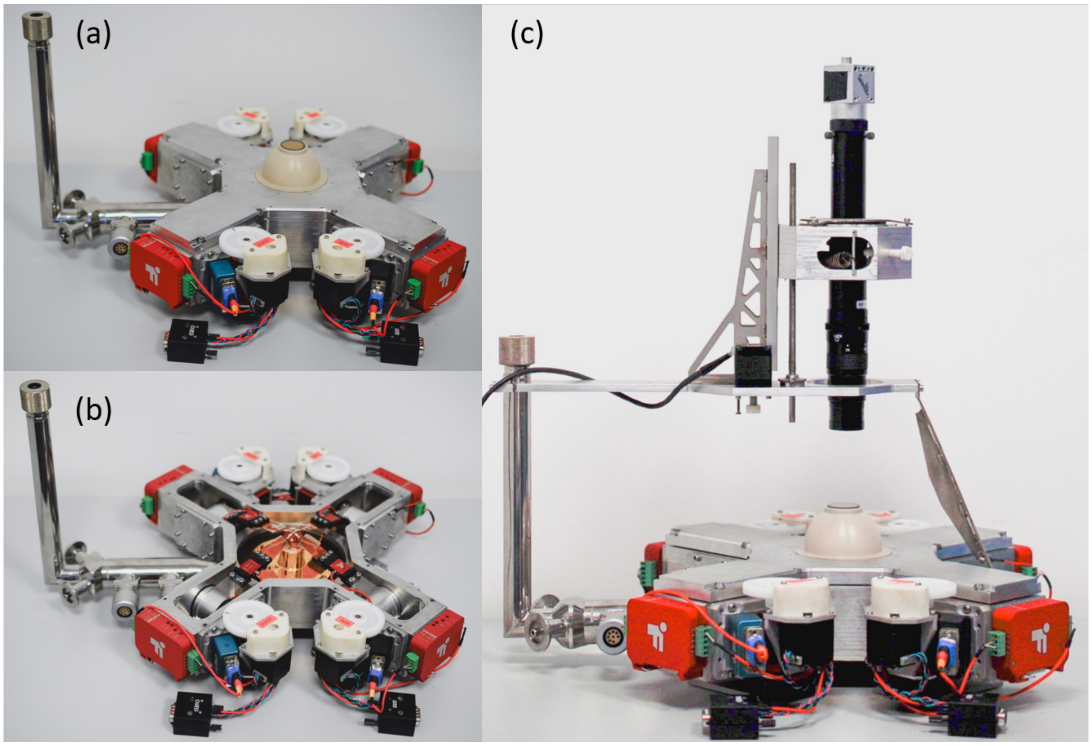

Supplementary Figure 1. **Pictures of the CryoBiaS device.** Global picture of the device with (a) and (b) without vacuum-tight cover. The device is enclosed in a vacuum chamber comprising the CryoVac cryostat and the different elements of the tensile stress device: deformable substrate, load cells and motorized wheel. The motors and reductors are outside the vacuum chamber. (c) The sample can be view through the optical window of the dome by a camera positioned above the sample, and can be used to estimate in-plane deformations using Digital Image Correlation method.

The cross-shaped cover is topped with a 300μm-thick dome made out of Polyetheretherketone (PEEK), that ensures good x-ray transmission at the 8keV working energy used here. An optical window is glued on top of the PEEK dome for optical measurements. The four perpendicular branches of the CryoBiaS device are equipped with motors and reductors that drive the cylindrical wheels inside the CryoBiaS chamber. The four branches of the 125μm-

thick polyimide cross-shaped deformable substrate are connected to each of the four wheels on all branches through calibrated load cells that measure the applied force on each branch independently during the experiment. The sample is glued in the center of the deformable substrate that lies on the cold finger of the cryostat in the center of the chamber. An optical Basler camera can be mounted on the device with appropriate optics to follow in-situ sample deformation through the optical window of the dome. This camera is used for the Digital Image Correlation (DIC) measurements presented in the following.

2. Characterization of strain in uniaxial and equibiaxial modes

The real sample elastic strain components are best measured by XRD on the sample of interest. However, the in-plane strain components can also be obtained by Digital Image Correlation (DIC) with a larger field of view to characterize the deformable substrate deformations.

In-plane strain components obtained by Digital Image Correlation (DIC) on the bare substrate: DIC measurements can be performed using a white paint speckle deposited on the bare substrate (see Suppl. Fig. 2(a)).

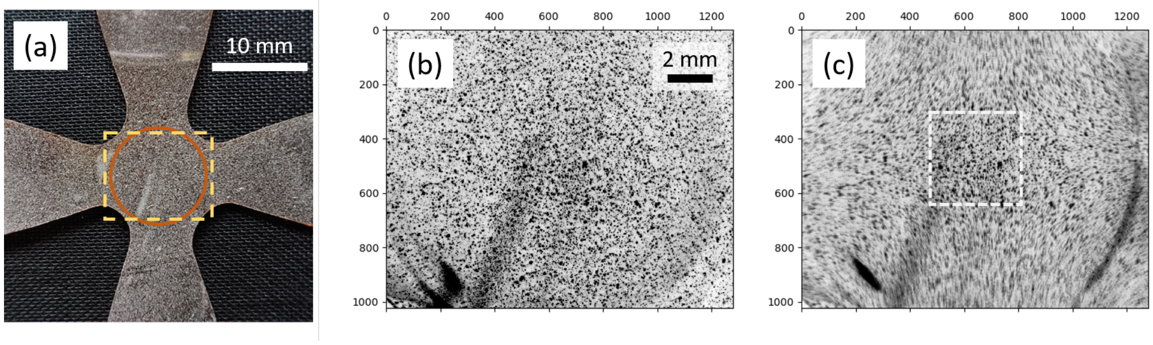

Supplementary Figure 2. **Paint speckle deposited on the bare deformable substrate for DIC.** (a) Large view of the cross-shaped deformable substrate. The paint speckle is visible as white dots on the surface. The orange circle represents the cryostat cold finger dimension on which the kapton substrate lies. The yellow dotted square represents the field-of-view seen by the Basler camera through the optics. (b) Typical image taken by the Basler camera. The black dots are the paint speckles that are tracked by DIC. (c) Sum of all images taken during uniaxial deformation of the substrate. The elongation of the paint speckles can be used to image the displacement field. The deformations are homogeneous in the 2.5×2.5 mm^2 central square zone, where the sample is placed.

The paint speckles are detected by the optical Basler camera through dedicated optics, as shown in Suppl. Fig. 2(b). We then used Correli^{STC} software to retrieve the two normal strain components ε_{11} and ε_{22} (directions 1 and 2 are along the machine axis and correspond to the vertical and horizontal directions of the images detected by the camera), as well as the shear strain component ε_{12}. We present DIC results for both uniaxial and equibiaxial tensile stresses (at room temperature here). We took 10 images per applied force in all cases, making 0.25kg (resp. 0.5kg) force steps in the case of the uniaxial (resp. equibiaxial) test. The displacement field can be imaged by summing all images of the test, as depicted in Suppl. Fig. 2(c). The normal and shear strain components obtained by DIC in the central

zone of the images are presented for uniaxial and equibiaxial tests in Suppl. Fig. 3.

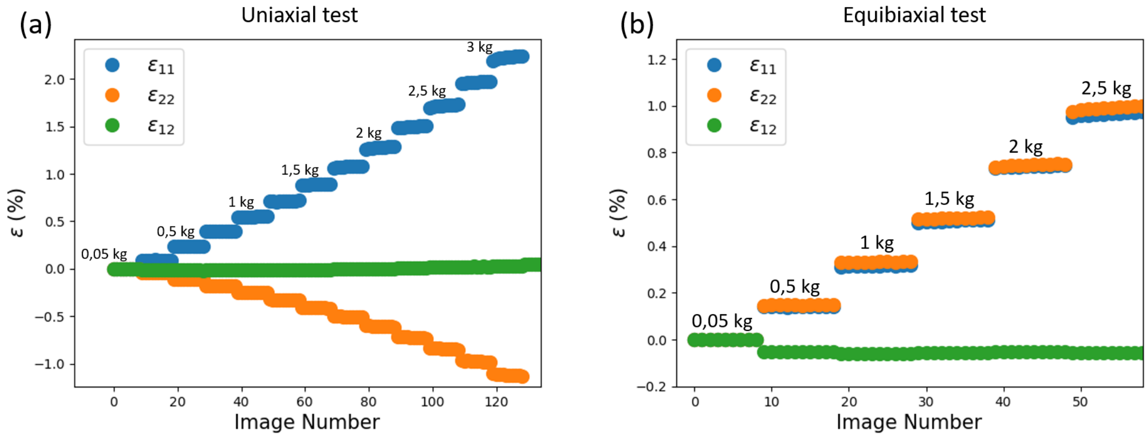

Supplementary Figure 3. **In-plane normal and shear strain components as extracted by DIC analysis.** Strain components obtained (a) for uniaxial test, making 0.25kg force steps and (b) for equibiaxial test, making 0.5kg force steps.

In the uniaxial test ε_{11} increases up to $\sim$2.23% at an applied force of 3kg, while ε_{22} decreases down to $\sim$-1.12%, with a ratio $\frac{\varepsilon_{22}}{\varepsilon_{11}} \sim -0.5 \pm 0.05$ during the test. The shear component is extremely small, with $|\varepsilon_{12}| < 0.04\%$. The shear component can thus be neglected.

In the equibiaxial test, the two normal strain components increase similarly up to $\sim$ 1% at 2.5kg, with a ratio $\frac{\varepsilon_{22}}{\varepsilon_{11}} \sim 1.03 \pm 0.02$ and a shear strain $\varepsilon_{12} = -0.06\% \pm 0.01\%$. Again, the shear strain is negligible compared to the normal strain components.

Direct measurement of shear in sample 1 by XRD: When a single crystal like Sample 1 is glued on the cross, it is possible to get the strain components directly from the XRD data, by following three non-collinear Bragg reflections. In the main text, we use the 0 16 0, 0 16 1 and 1 15 0 to retrieve the three lattice parameters a, b and c of $TbTe_3$ as a function of applied force in sample 1, as shown in Fig. 2(b) of the main text, and further described in the following section I B. This allows to get the normal strain components if shear can be neglected. The shear value can be obtained directly by analyzing the position of the three non-collinear Bragg reflections in reciprocal space, as depicted in Suppl. Fig. 4(a).

The determination of α^*, β^* and γ^* in Sample 1, extracted from the same data as presented in Fig. 2(b) of the main text show that their variation is within the error bars. We find the following average values: $\alpha^* = 89.94 \pm 0.08^o$, $\beta^* = 89.89 \pm 0.14^o$ and $\gamma^* = 89.91 \pm 0.06^o$. Shear is thus considered negligible in the conditions of the experimental data shown here.

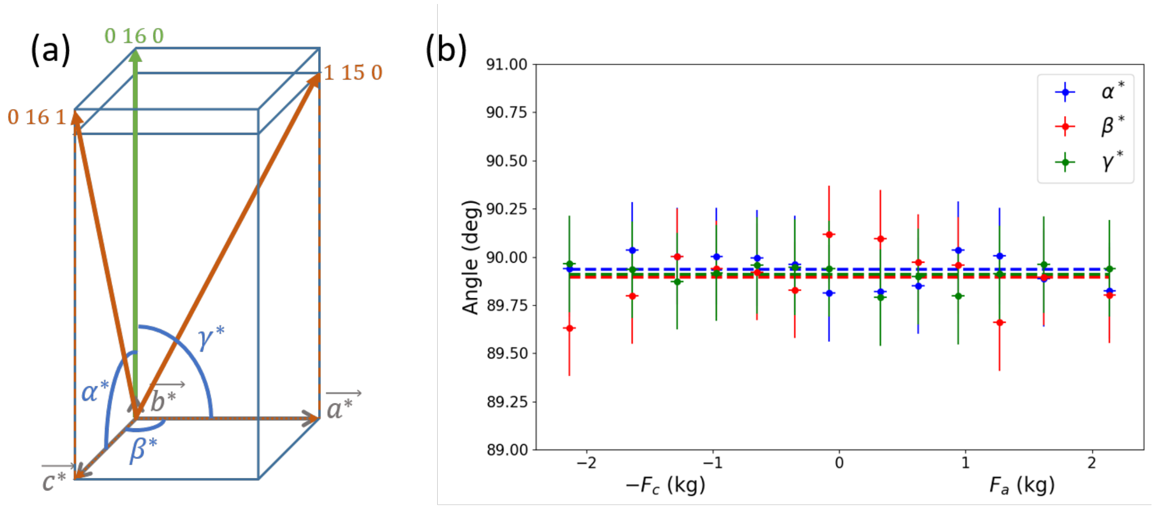

Supplementary Figure 4. **Analysis of shear in Sample 1 by XRD.** (a) Sketch of the three non collinear Bragg reflections recorded during the XRD experiment in the reciprocal cell (not to scale). $\frac{1}{16}(0\ 16\ 0)$ directly gives $\vec{b^*}$. The two non-specular reflections 0 16 1 and 1 15 0 can be projected onto the $(\vec{a^*}, \vec{c^*})$ plane by subtracting the 0 16 0 and $\frac{15}{16}(0\ 16\ 0)$ wavevectors respectively to get the 0 0 1 and 1 0 0 reciprocal vectors respectively. The three angles of the reciprocal unit cell α^*, β^* and γ^* can then be obtained by the scalar products of the three reciprocal base vectors. (b) α^*, β^* and γ^* computed as a function of applied forces F_a and $-F_c$ for the same data as presented in Fig. 2(b) of the main text in Sample 1.

B. Analysis of XRD data

1. *Determination of lattice parameters from XRD data*

In both laboratory and synchrotron experiments, the biaxial tensile device was mounted at the center of rotation of a diffractometer used in Eulerian 4-circle geometry. In both cases, detection was performed with a 2D pixel detector. At LPS, we used a Timepix detector from ASI, and a Merlin detector from Quantum Detectors for the data taken at the DIFFABS beamline of SOLEIL synchrotron, both made of a grid of 516x516 square pixels (pixel size : $p = 55\mu$m). The sample-detector distance D was 82cm at LPS and 65cm at SOLEIL.

We work in the fixed laboratory frame, defined with its origin on the sample $(\vec{x}, \vec{y}, \vec{z})$. The detector is mounted on an arm that can be moved around the sample by two rotations (δ, γ) as depicted in Suppl. Fig. 5a. When the detector is in the direct beam $(\delta = 0, \gamma = 0)$, each pixel of the detector array $M(i, j)$ has coordinates $\vec{r}_M = (x_M, y_M, z_M)$:

$$\vec{r}_M = \begin{cases} x_M &= D \\ y_M &= i.p \\ z_M &= j.p \end{cases} \tag{1}$$

The pixel hit by the center of the direct beam is taken as reference with $i = j = 0$.

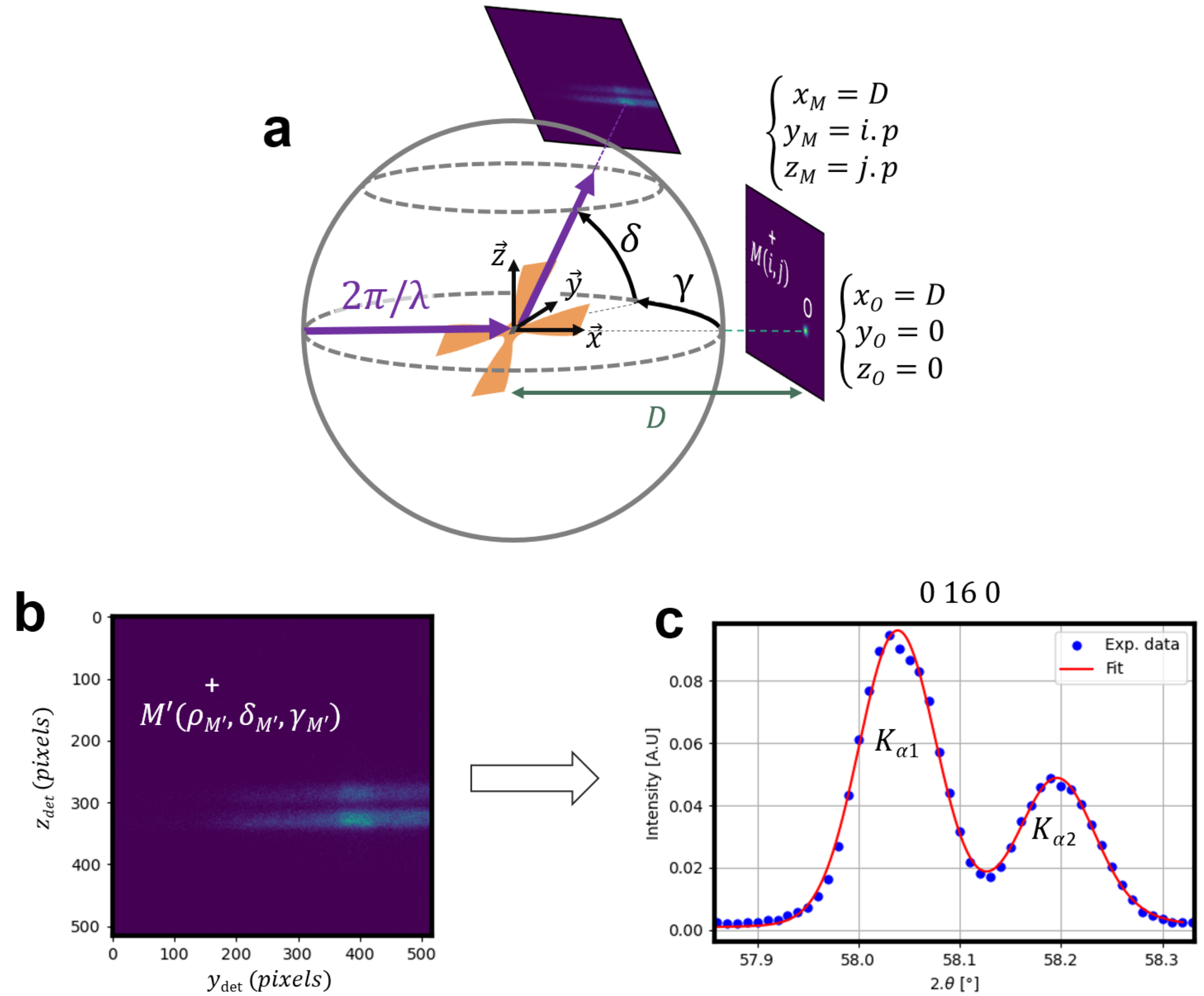

Supplementary Figure 5. **Diffraction geometry and conversion of XRD images into $I(2\theta)$ plots.** (a) Sketch of the diffraction geometry in reflection condition. The Ewald sphere depicts the elastic diffraction process, which has a radius $2\pi/\lambda$ defined by the beam wavelength λ. The sample, glued on the cross and installed into the biaxial tensile deformation device, is placed at the center of this sphere, which is the center of rotation of the diffractometer used in 4-Circles Eulerian geometry. The 2D detector is placed at a distance D from the sample, and is carried by an arm that can rotate around the sample with 2 rotations δ and γ moving in the vertical and horizontal planes respectively. The pixels of the detector are assigned Cartesian coordinated in the fixed laboratory frame and are computed for each position (δ, γ) of the detector arm, taking the direct beam position on the detector as the reference point O. Any other pixel indexed (i, j) on the detector has non-zero coordinates. (b) Typical image obtained on a Bragg reflection by summing all images on the rocking curve. (c) A 2θ value is computed for each pixel of the detector, as described in the text, and allows to convert the sum image shown in (b) into a $I(2\theta)$ graph.

When the detector is moved to any set of (δ, γ) angles, the same pixel (i, j) is changed in the point M' with coordinates $\vec{r}_{M'} = \begin{pmatrix} x_{M'} \\ y_{M'} \\ z_{M'} \end{pmatrix}$ such that :

$$\vec{r}_{M'} = R_z(\gamma) \cdot R_y(-\delta) \cdot \vec{r}_M \tag{2}$$

that can be in turn converted into spherical coordinates $\vec{r}_{M'} = (\rho_{M'}, \delta_{M'}, \gamma_{M'})$ such that :

$$\vec{r}_{M'} = \begin{cases} \rho_{M'} &= \sqrt{x_{M'}^2 + y_{M'}^2 + z_{M'}^2} \\ \delta_{M'} &= \sin^{-1}\left(\frac{z_{M'}}{\rho_{M'}}\right) \\ \gamma_{M'} &= \tan^{-1}\left(\frac{y_{M'}}{x_{M'}}\right) \end{cases} \tag{3}$$

Finally, we compute the $2\theta_{M'}$ value of M' pixel thanks to the equation :

$$\cos(2\theta_{M'}) = \cos(\delta_{M'}) \, . \cos(\gamma_{M'}) \tag{4}$$

After doing so for all pixels of the detector, we average the intensities recorded by pixels sharing the same 2θ value within 0.01 degree, and get the $I(2\theta)$ curve for each recorded image.

This process was applied to get the intensity profiles as a function of 2θ values from the images recorded on the 3 Bragg reflections 0 16 0, 1 15 0 and 0 16 1, as shown in Suppl. Fig. 5b-c. In the laboratory experiment, we get two peaks due to the two K_{α_1} and K_{α_2} components of the x-ray beam ($\lambda_{K_{\alpha_1}} = 1.54060$ Å, $\lambda_{K_{\alpha_2}} = 1.54443$ Å). The laboratory data were fitted with a double Voigt function :

$$F(x) = V_1(x, \mu_1, A_1, \sigma_1, \gamma_1) + V_2(x, \mu_2, A_2, \sigma_2, \gamma_2) + B \tag{5}$$

where B is a constant to account for background signal and V_1 and V_2 are Voigt functions $V(x, \mu, A, \sigma, \gamma)$ with amplitude A defined as the convolution of a Gaussian function $G(x, \mu, \sigma)$ (with center $x = \mu$ and width σ) and a Lorentzian function $L(x, \mu, \gamma)$ (with center $x = \mu$, width γ).

The fit of the intensity profiles $I(2\theta)$ gives the positions μ_1 and μ_2 of the two peaks associated to K_{α_1} and K_{α_2}. The Bragg law is then used to get the lattice constants for each set of forces F_a and F_c from the first peak generated by the K_{α_1} component. The $\|\vec{b}\|$ lattice parameter is first obtained from $2\theta_{0,16,0}$ and knowing it, the $\|\vec{a}\|$ and $\|\vec{c}\|$ lattice parameters are obtained from $2\theta_{1,15,0}$ and $2\theta_{0,16,1}$ respectively, assuming the crystal keeps an orthorhombic unit cell. The evolution of the 3 lattice parameters can then be plotted as a function of the applied forces, as shown in Fig.2b of the main article.

The same procedure was applied to get lattice parameters on another $TbTe_3$ sample (Sample 3), measured at the DIFFABS beamline of SOLEIL synchrotron. There, the x-ray beam energy was set to 8.8 keV with a Si(111) monochromator, providing an energy resolution $\Delta E/E \sim 1.6 \cdot 10^{-4}$, and the beam size was 266×154 µm^2 (H×V)

at sample position. Detection was performed with a 2D pixel detector with 55 μm pixel size (Merlin detector from Quantum Detectors) located ~65 cm downstream of the sample. The crystal was also glued on a Kapton substrate and forces F_a and F_c were applied along the $\vec{a}$ and $\vec{c}$ directions of the crystal with the biaxial deformation device at T=350 K. In this case the 3 non-collinear Bragg peaks 0 16 0, 0 18 2 and 2 18 0 were followed as a function of applied force F_c while a force $F_a = 0.69kg$ was applied along $\vec{a}$. The intensity profiles $I(2\theta)$ of the three Bragg peaks were retrieved using the same calculations as in Suppl. Eq. 1-5 and are shown in Suppl. Fig. 6a-c.

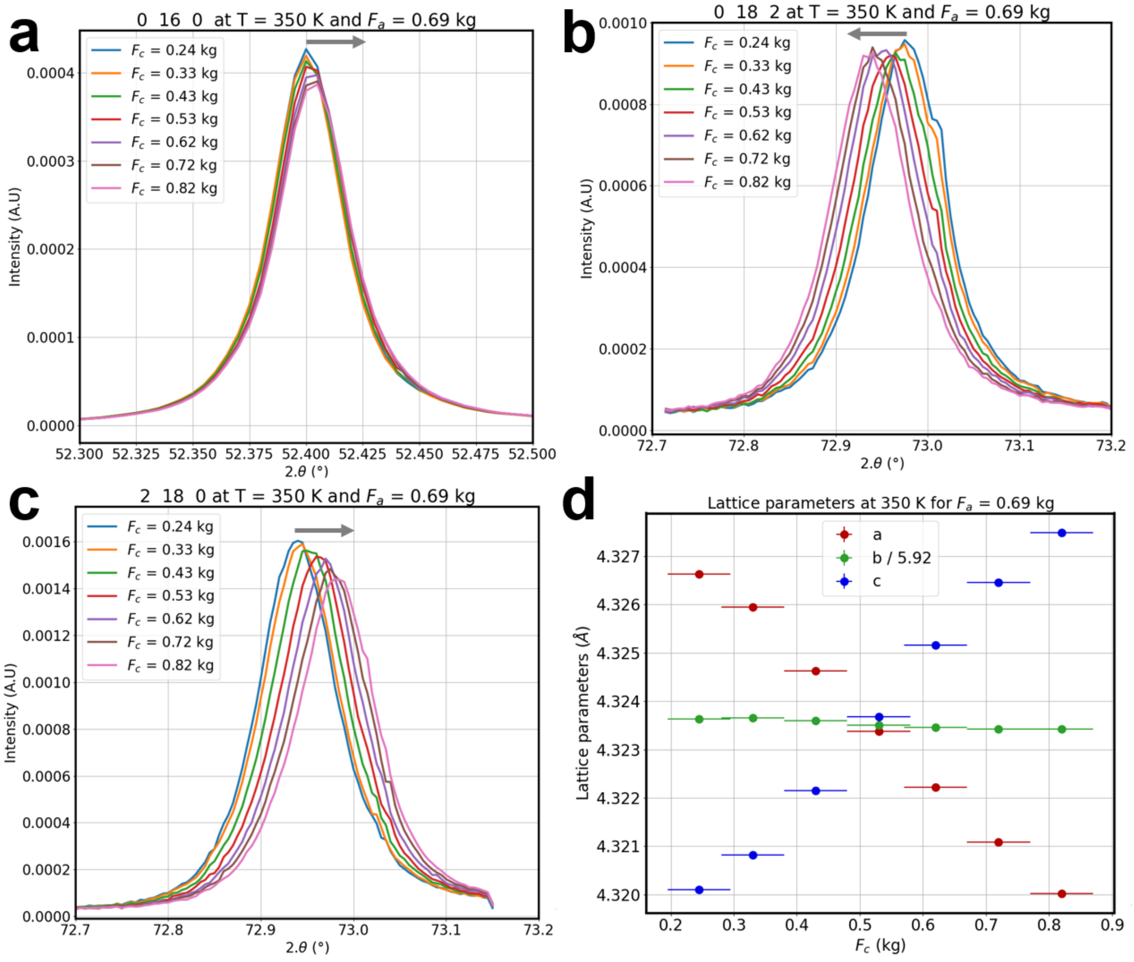

Supplementary Figure 6. **Analysis of lattice parameters from 3 non-collinear Bragg peaks on sample 3 at synchrotron SOLEIL.** Rocking scans measured as a function of increasing force F_c at constant $F_a = 6.75N$ at 350K on Sample 3 on 3 non-collinear Bragg reflections : (a) the 0 16 0; (b) the 0 18 2 and (c) the 2 18 0. (d) Evolution of the 3 lattice parameters a, b and c computed with the positions of the measured Bragg peaks shown in (a)-(c) as a function of F_c, assuming that all lattice angles are kept to 90 deg.

These peaks were fitted using Suppl. Eq. 5 with a single Voigt function, and the three lattice parameters computed in the same way as described previously. The evolution of the lattice parameters are shown in Suppl. Fig. 6d.

At very low forces F_c, $a > c$ because we started this measurement with a non-zero force $F_a = 0.69kg$. When F_c is increased, c increases linearly with a slope ~0.22%/kg while a decreases linearly at a rate ~-0.25%/kg. As for Sample 1 in the main text, the variations of a and c are found to be very similar, and very linear with the high resolution of

XRD measurement in the synchrotron. This confirms the in-plane Poisson ratio $\nu_{ac} \sim 1$.

The evolution of the $b/5.92$ parameter is also shown on Suppl. Fig. 6d (b is divided by 5.92 to be compared to the variations of a and c on the same graph). It varies by ∼0.008%/kg in these conditions, *i.e.* 30 times less compared to the variations of the in-plane parameters a and c. Here $\nu_{ab}^{Sample2} \sim 0.03$ instead of $\nu_{ab}^{Sample1} \sim 0.1$ reported for Sample 1 in the main article.

2. Analysis of CDW peaks

The CDW reflections were recorded with the same geometry and detector as shown in Suppl. Fig. 5a. Rocking curves were measured by recording an image at various rocking angles ω around the peak maxima. As the CDW satellite intensities are weak, the background (due to scattering by surrounding elements, mainly air) has to be subtracted to get a significant signal/noise ratio. The CDW diffraction signal was selected in a Region of Interest (called ROI) defined in such a way that only the reflection associated to the K_{α_1} component was kept. A second region of interest having the same size as ROI was defined in a region of the detector with no CDW diffraction signal (called BCKG), thus containing only 'background' signal, coming from other scattering processes which are homogeneous and randomly distributed on the detector pixels. We thus get a corrected intensity signal $I_{corr}(\omega)$ for each image taken at each angle of the rocking curve on the CDW peak, that reads :

$$I_{corr}(\omega) = \sum_{(i,j)\in ROI} I(i,j) - \sum_{(i,j)\in BCKG} I(i,j) \tag{6}$$

where (i,j) are pixel coordinates on the detector, and $I(i,j)$ is the recorded intensity on each pixel (i,j). We thus obtain the rocking curves with corrected intensities as shown in Suppl. Fig. 7 and shown in Fig. 3 of the main article.

Finally, the integrated intensities for each set of force F_a and F_c, used to plot Fig.4a of the main article, was obtained by direct integration of $I_{corr}(\omega)$ along ω :

$$I_{F_a,F_c} = \sum_{\omega} I_{corr}(\omega) \tag{7}$$

The associated errors bars $\Delta I_{F_a,F_c}$ were set to $\sqrt{I_{F_a,F_c}}$.

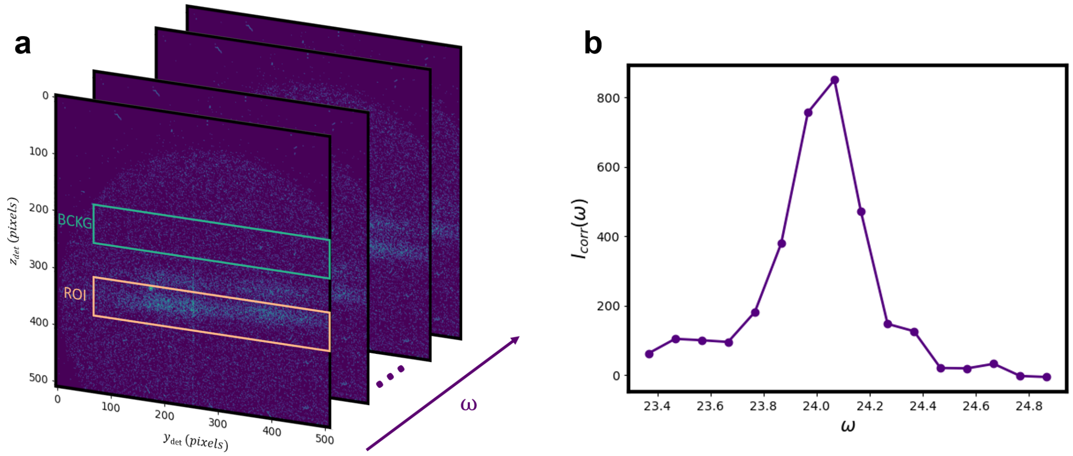

Supplementary Figure 7. **Analysis of diffraction images taken on the CDW peaks.** (a) Images taken at several ω rocking angles around the CDW peak. The signal appears at the same position as the direct beam when detector is at zero lifting angles. Two regions of interest with same size are defined on the detector : one with the relevant signal coming from the CDW reflection (ROI) and one with only background signal (BCKG). (b) Rocking curve obtained when subtracting the background intensity from the signal intensity for each image of the rocking curve (Suppl. Eq. 6).

C. Transport measurements

1. *Resistance measurement in the Montgomery configuration*

To perform the transport measurements, 4 contacts were deposited on the 4 corners of the rectangular samples by cold soldering, as depicted in Suppl. Fig. 8.

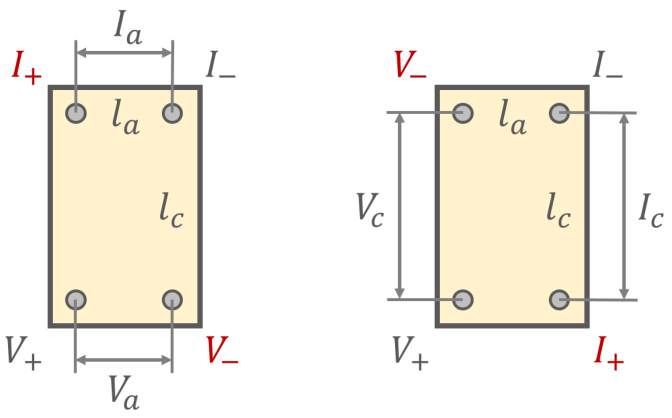

Supplementary Figure 8. **Sketch of contacts geometry for Montgomery transport measurements.** Two configurations are shown, to perform transport measurement along the $\vec{a}$ (left drawing) and $\vec{c}$ (right drawing) crystallographic directions of the sample. Two contacts I_+ and I_- are used for current injection (I_a along $\vec{a}$, I_c along $\vec{c}$) and two others V_+ and V_- for voltage measurement (V_a along $\vec{a}$, V_c along $\vec{c}$). The distance between contact is called l_a along $\vec{a}$ and l_c along $\vec{c}$. Switching from one configuration to another is ensured by an automatic procedure that inverts the I_+ and V_- contacts (shown in red on the drawing).

The total measured voltage is the sum of thermal voltage V^{th} and sample voltage V^S. To get the sample resistance alone, two measurements are performed with opposite currents. The voltages are measured for both current signs, and we get two voltage measurements V_{aa+} and V_{aa-} for the two current directions $+I_a$ and $-I_a$ when measuring

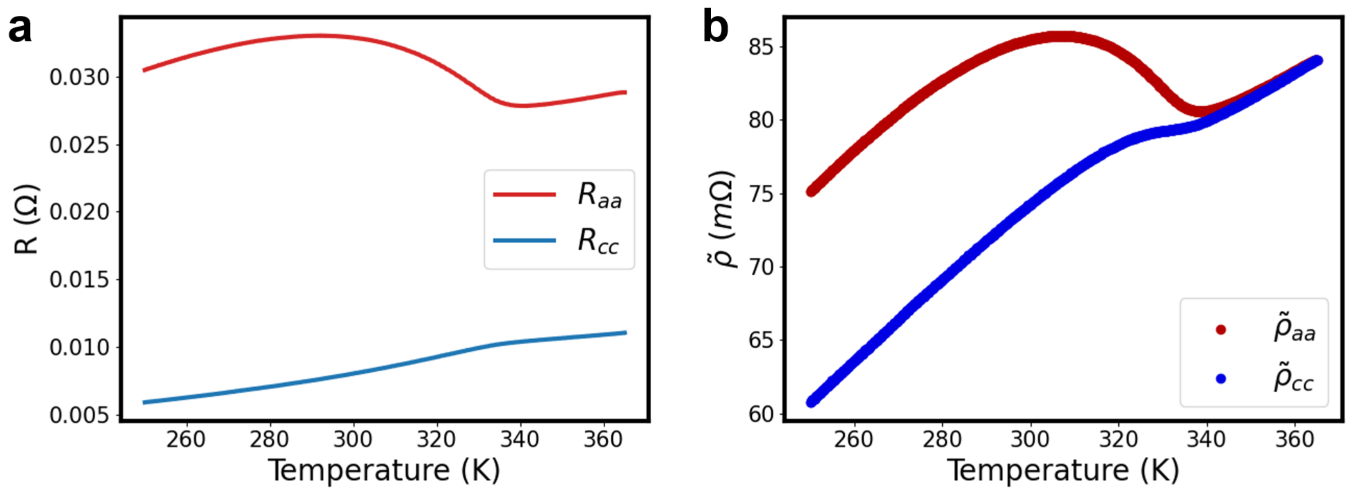

Supplementary Figure 9. **Resistance and resistivities alonf $\vec{a}$ and $\vec{c}$ in $TbTe_3$, without force.** (a) Sample resistances R_{aa} and R_{cc} as a function of temperature, as obtained using Suppl. Eq. 9. (b) corresponding resistivities divided by sample thickness as obtained using Suppl. Eq. 10.

along the $\vec{a}$ crystallographic direction of the sample (respectively V_{cc+} and V_{cc-} along $\vec{c}$ for currents $+I_c$ and $-I_c$). We thus get the following total resistances R_{aa+} and R_{aa-} for positive and negative currents along $\vec{a}$ (resp. R_{cc+} and R_{cc-} along $\vec{c}$):

$$\begin{cases} R_{aa+} = \frac{V_{aa+}}{I_a} = \frac{V_{aa}^{th}+V_{aa}^{S}}{I_a} \\ R_{aa-} = \frac{V_{aa-}}{-I_a} = \frac{V_{aa}^{th}-V_{aa}^{S}}{-I_a} \end{cases} ; \quad \begin{cases} R_{cc+} = \frac{V_{cc+}}{I_c} = \frac{V_{cc}^{th}+V_{cc}^{S}}{I_c} \\ R_{cc-} = \frac{V_{cc-}}{-I_c} = \frac{V_{cc}^{th}-V_{cc}^{S}}{-I_c} \end{cases} \tag{8}$$

and thus get the sample resistances along $\vec{a}$ and $\vec{c}$ with the following equations:

$$\begin{cases} R_{aa} = \frac{R_{aa+}+R_{aa-}}{2} = \frac{1}{2}\left(\frac{V_{aa+}-V_{aa-}}{I_a}\right) \\ R_{cc} = \frac{R_{cc+}+R_{cc-}}{2} = \frac{1}{2}\left(\frac{V_{cc+}-V_{cc-}}{I_c}\right) \end{cases} \tag{9}$$

The injected currents are generally ∼1mA. Typical resistances R_{aa} and R_{cc} obtained with this method in Sample 1 are shown in Suppl. Fig. 9a for temperatures ranging between 250K and 365K. Both resistance curves show a linear behaviour above T_c and a resistance increase when the sample gets into the CDW phase below T_c.

2. Conversion of resistances into resistivities

The resistivities ρ_{aa} and ρ_{cc} can be obtained from R_{aa} and R_{cc} in the Montgomery geometry using some sets of equations described in [1, 2]. Simplified expressions have been used here, following the work of Ong and Brill in $NbSe_3$ [3], and are recalled in the following.

After getting the sample resistances along $\vec{a}$ and $\vec{c}$, R_{aa} and R_{cc}, using the method presented in the previous

paragraph and in particular Suppl. Eq. 9, one can compute the resistivities ρ_{aa} and ρ_{cc} with the following formulae, in the case of thin samples:

$$\begin{cases} \rho_{aa} &= H(x).d.R_{aa}.\frac{x}{l_a/l_c} \\ \rho_{cc} &= H(x).d.R_{aa}.\frac{l_a/l_c}{x} \end{cases} \tag{10}$$

where:

- d is the sample thickness

- l_a and l_c the distance between contacts along $\vec{a}$ and $\vec{c}$ crystallographic directions of the sample

- $x = A + B\ln r + C\left(\ln r\right)^2 + D\left(\ln r\right)^3 \ldots$
  with $A = 0.997229, B = 0.160697, C = 1.231448 \cdot 10^{-2}, D = -3.79740 \cdot 10^{-4}$
  and $r = \frac{R_{aa}}{R_{cc}}$

and $H(x)$ is the function :

$$H(x) \equiv \frac{\pi}{4}\ln\frac{1+2q+2q^4\cdots}{1-2q+2q^4\cdots} \;\;,\;\; q = e^{-\pi x} \tag{11}$$

that can be simplified into:

$$H(x) = \frac{\pi}{8}\sinh\left(\frac{\pi}{x}\right) \tag{12}$$

As the sample thickness d is not precisely measured here, we work with the rescaled resistivities $\tilde{\rho}_{aa} = \frac{\rho_{aa}}{d}$ and $\tilde{\rho}_{cc} = \frac{\rho_{cc}}{d}$ in the manuscript. However, we can get a good estimation of the sample thickness by comparing our values of ρ_{aa} and ρ_{cc} to the ones reported in the literature [4, 5], and find that Sample 1 has a thickness $\sim$2.5μm and Sample 2 a thickness $\sim$10μm.

The resistivities ρ_{aa} and ρ_{cc} calculated with these equations from the resistances R_{aa} and R_{cc} shown in Suppl. Fig. 9a, are shown in Suppl. Fig. 9b.

Note that the anisotropy does not dependent on sample thickness: $\frac{\rho_{aa}}{\rho_{cc}} = \frac{\tilde{\rho}_{aa}}{\tilde{\rho}_{cc}}$.

3. Determination of resistivity jumps

In the main article, we analyze the evolution of the resistivity jumps $\Delta\tilde{\rho}_{aa}$ and $\Delta\tilde{\rho}_{cc}$. To extract these quantities from the resistivity curves $\tilde{\rho}_{aa}$ and $\tilde{\rho}_{cc}$, we first perform a linear fit of the resistivity in the normal state (above T_c) and call these components $\tilde{\rho}_{aa}^N$ and $\tilde{\rho}_{cc}^N$ (see Suppl. Fig. 10a and c). We then subtract it from $\tilde{\rho}_{aa}$ and $\tilde{\rho}_{cc}$ to get the corrected resistivity curves $\tilde{\rho}_{aa}^c = \tilde{\rho}_{aa} - \tilde{\rho}_{aa}^N$ and $\tilde{\rho}_{cc}^c = \tilde{\rho}_{cc} - \tilde{\rho}_{cc}^N$ (see Suppl. Fig. 10b and d).

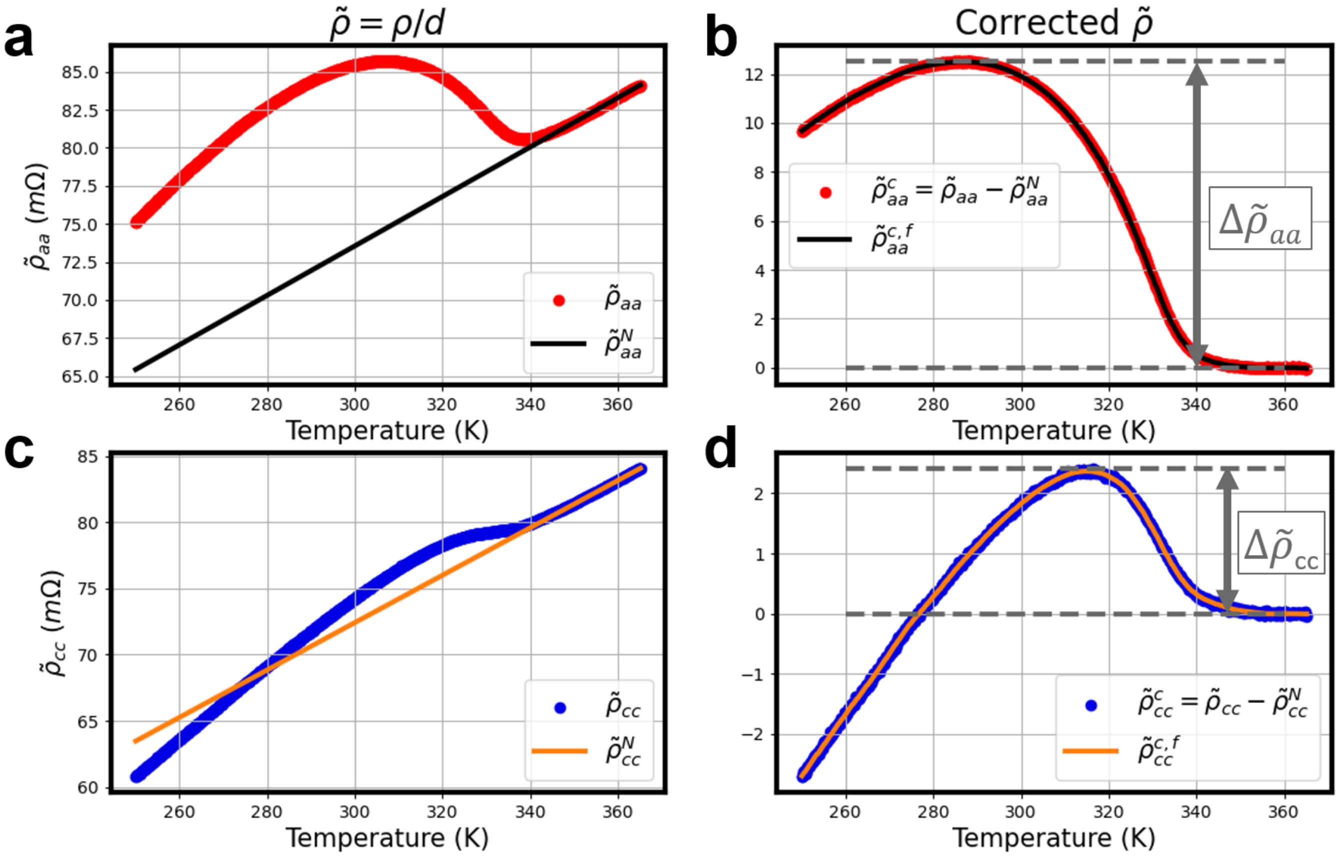

Supplementary Figure 10. **Method to obtain the resistivity jumps $\Delta\tilde{\rho}_{aa}$ and $\Delta\tilde{\rho}_{cc}$.** (a) $\tilde{\rho}_{aa}$ as obtained from the temperature-dependent resistance R_{aa} with the method described in Section I C 2. The linear fit of the normal state resistivity $\tilde{\rho}_{aa}^N$ is plotted in black. (b) Plot of corrected resistivity $\tilde{\rho}_{aa}^c$ as a function of temperature. The resistivity jump $\Delta\tilde{\rho}_{aa}$ corresponds to the maximum value of $\tilde{\rho}_{aa}^c$. $\tilde{\rho}_{aa}^{c,f}$ is the filtered corrected resistivity, using the method described in the text. (c) and (d) Same as (a) and (b) applied to resistivity data along $\vec{c}$.

We introduce here the filtered corrected resistivities $\tilde{\rho}_{aa}^{c,f}$ and $\tilde{\rho}_{cc}^{c,f}$ that are used in the following to obtain precise values of T_c. A Savitzky-Golay algorithm has been applied using a second-degree polynomial over ∼100 data points to damp experimental point fluctuations, mainly coming from the temperature instabilities over few mK. This is particularly important to get cleaner first and second derivatives of $\tilde{\rho}_{aa}$ and $\tilde{\rho}_{cc}$ for the determination of T_c (see Section I C 4). The anisotropy $\frac{\rho_{aa}}{\rho_{cc}}$ was also filtered using the same procedure in the following.

The resistivity jumps $\Delta\tilde{\rho}_{aa}$ and $\Delta\tilde{\rho}_{cc}$ are the maxima of $\tilde{\rho}_{aa}^c$ and $\tilde{\rho}_{cc}^c$ respectively.

4. *Determination of* T_c

To determine T_c, we used the resistivity data $\tilde{\rho}^c_{aa}$, $\tilde{\rho}^c_{cc}$ and the anisotropy $\frac{\rho^c_{aa}}{\rho^c_{cc}}$ as a function of temperature. We first take the first derivative of these three quantities (see Suppl. Fig 11a,c,e), as well as the first derivative of the filtered resistivities.

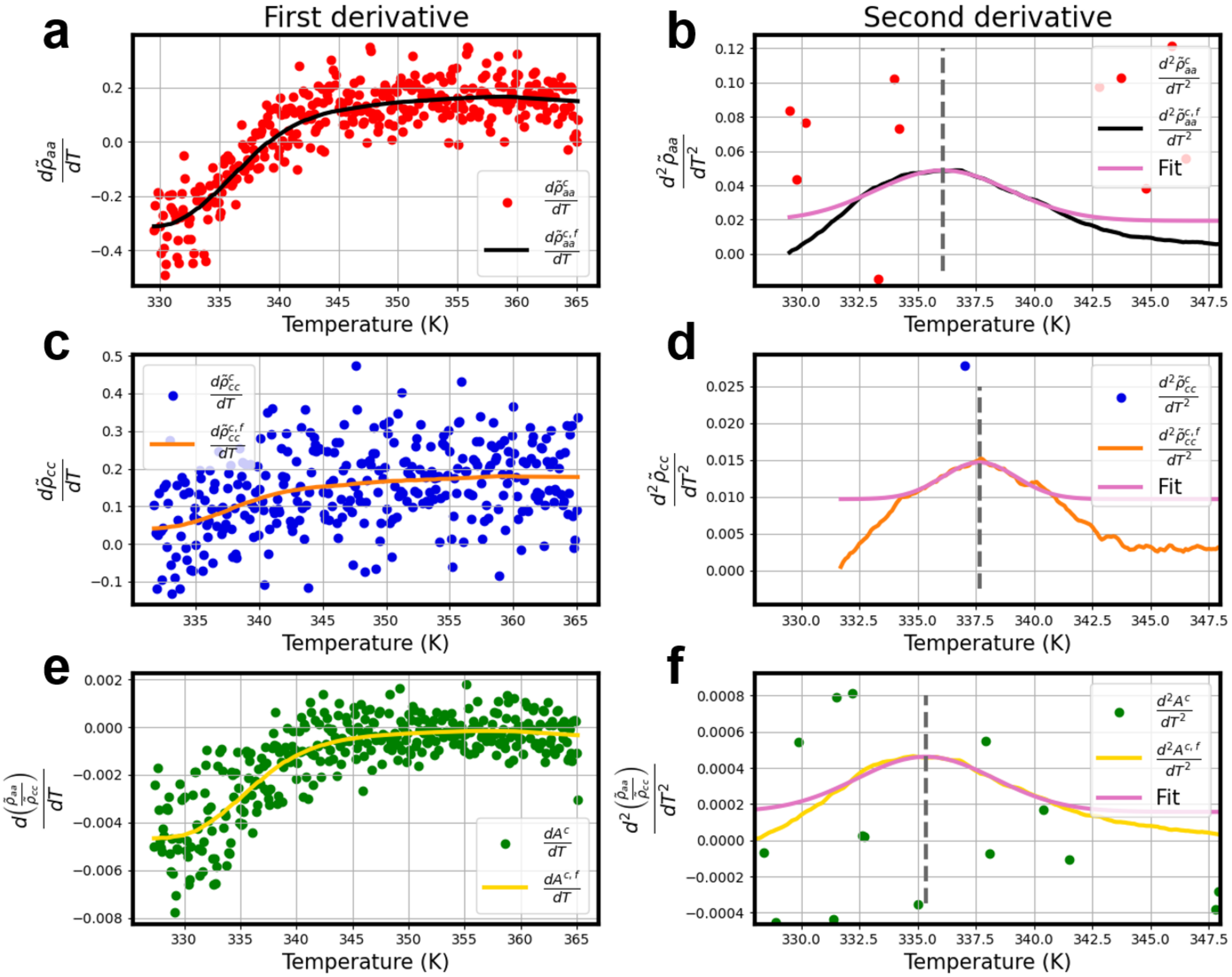

Supplementary Figure 11. **Method for T_c determination from transport data.** (a) First derivative of $\tilde{\rho}^c_{aa}$ (red dots) and $\tilde{\rho}^{c,f}_{aa}$ (black curve) as a function of temperature. (b) Second derivative of the same quantities and fit of $\frac{d\tilde{\rho}^{c,f}_{aa}}{dT}$ (pink curve). (c) and (e) Same as (a) for $\tilde{\rho}^c_{cc}$ and anisotropy. (d) and (f) Same as (b) for $\tilde{\rho}^c_{cc}$ and anisotropy.

The derivative of filtered data is completely in agreement with the non-filtered data, but are much less noisy. On all three first derivatives $\frac{d\tilde{\rho}^c_{aa}}{dT}$, $\frac{d\tilde{\rho}^c_{cc}}{dT}$ and $\frac{d\left(\frac{\rho^c_{aa}}{\rho^c_{cc}}\right)}{dT}$, a sigmoid-like curve appears, centered at T_c. To determine precisely the position of this inflection point and get T_c, we take the second derivative of these three quantities $\frac{d^2\tilde{\rho}^c_{aa}}{dT^2}$, $\frac{d^2\tilde{\rho}^c_{cc}}{dT^2}$ and $\frac{d^2\left(\frac{\rho^c_{aa}}{\rho^c_{cc}}\right)}{dT^2}$ (see Suppl. Fig 11b,d,f). Again, taking the second derivative on the filtered data is unavoidable to get clean and usable data. A Gaussian fit allows to get the position of T_c from the three sets of data. We thus obtained

three values of T_c for each experimental point corresponding to one set of applied forces. We plot the T_c obtained from each quantity $\tilde{\rho}^c_{aa}$, $\tilde{\rho}^c_{cc}$ and the anisotropy $\frac{\rho^c_{aa}}{\rho^c_{cc}}$ ass a function of a/c ratio in Suppl. Fig. 12.

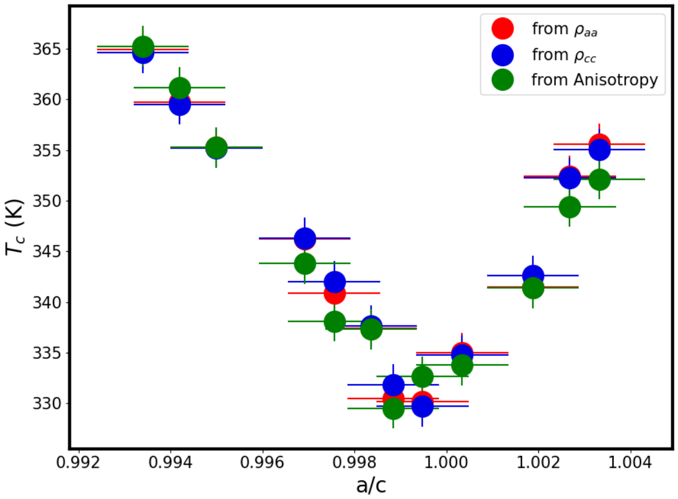

Supplementary Figure 12. **Variation of T_c for all transport data in Sample 1.** T_c obtained from each quantity $\tilde{\rho}^c_{aa}$ (red dots) $\tilde{\rho}^c_{cc}$ (blue dots) and the anisotropy $\frac{\rho^c_{aa}}{\rho^c_{cc}}$ (green dots).

The curves of T_c as a function of a/c ratio all behave the same within a couple of K. The final curve shown in the text (Fig. 4c of the main article) is the average of the three T_c shown here.

D. Additional results - Sample 2

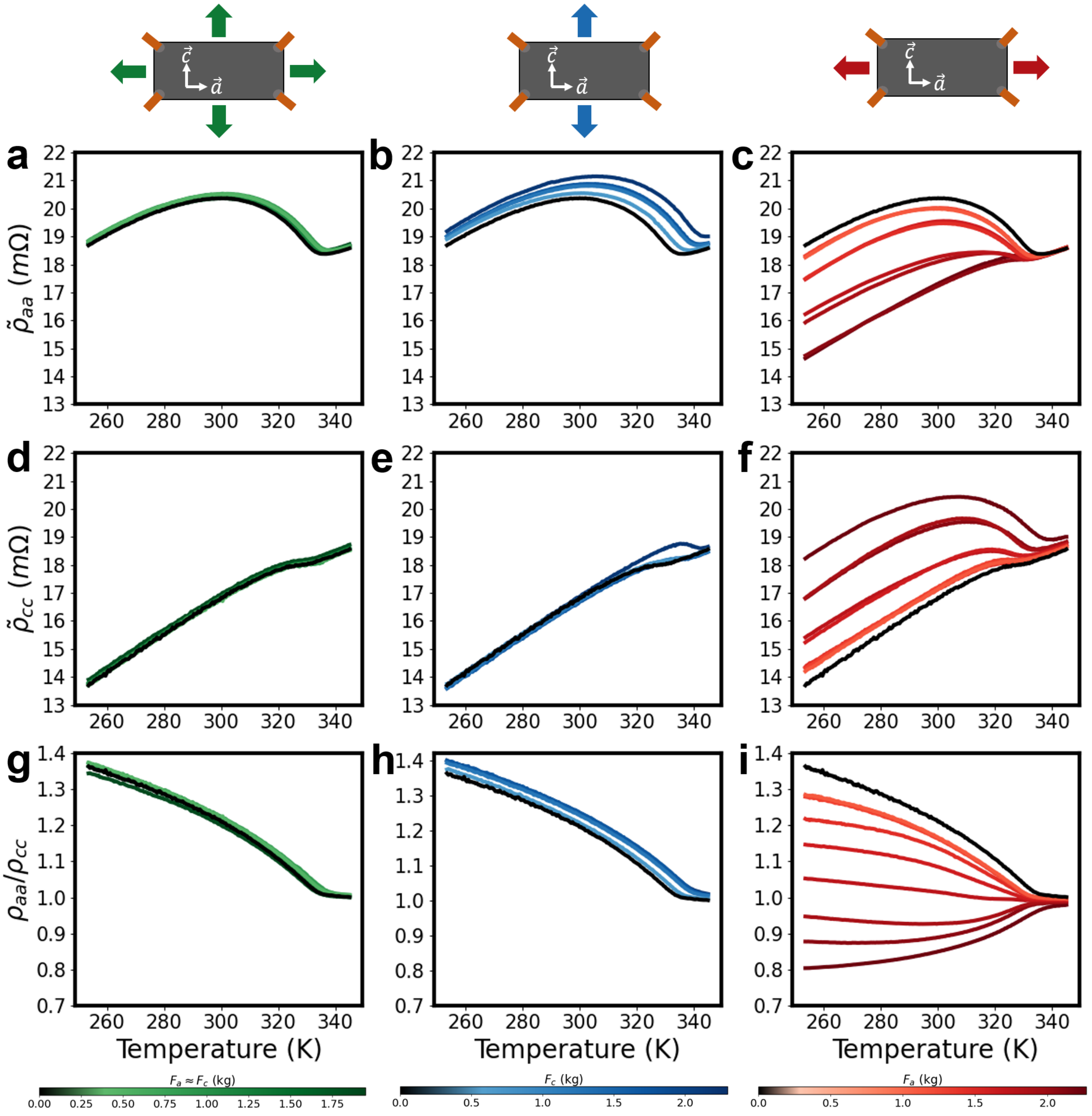

Supplementary Figure 13. **Transport measurement in Sample 2 for equibiaxial and uniaxial in-plane deformations.** (a), (d) Resistivities obtained from resistances along $\vec{a}$ and $\vec{c}$ using Eq. 10 to get $\tilde{\rho}_{aa}$ and $\tilde{\rho}_{cc}$ as a function of temperature in Sample 2 between 250K and 350K for equibiaxial forces $F_a = F_c$ ranging from 0 to 1.3kg (12.75N). (g) Corresponding anisotropies ρ_{aa}/ρ_{cc} as a function of temperature. (b),(e),(h) and (c),(f),(i) : same quantities as in (a),(d),(g) measured in the same Sample 2 for uniaxial forces along $\vec{a}$ and $\vec{c}$ respectively. The colorbars shown at the bottom of the columns are common for all figures of the same column, for either equibiaxial, uniaxial forces along $\vec{a}$ and uniaxial forces along $\vec{c}$, as depicted in the schematic drawings shown at the top of each column. The black curve of each graph corresponds to the pristine state.

The transport data obtained in Sample 2 are shown in Suppl. Fig. 13, for both equibiaxial and uniaxial forces, to

complement equibiaxial data presented in the main article for which no variation of resistivity (and hence anisotropy) are observed, contrary to the huge variations observed for uniaxial deformations. Here we show that Sample 2 displays the same behaviour as Sample 1, both for variation of T_c and $\Delta\tilde{\rho}$. Indeed, when Sample 2 is deformed along $\vec{c}$ (applied forces F_c, $F_a = 0$), $\Delta\tilde{\rho}_{aa}$, $\Delta\tilde{\rho}_c$ and anisotropy (Suppl. Fig. 13(b),(e),(h)) continuously shift to higher temperatures with no clear change of resistivity jump. On the contrary, when forces are applied to Sample 2 along $\vec{a}$ (applied force F_a, $F_c = 0$), the jump $\Delta\tilde{\rho}_{aa}$ (resp. $\Delta\tilde{\rho}_{cc}$) decreases (resp. increases) continuously as a function of increasing F_a, and hence the anisotropy is reversed (Suppl. Fig. 13(c),(f),(i)). The variations of T_c are detailed in the following.

The resistivity jumps corresponding to the data shown in Suppl. Fig. 13 have been analyzed with the method presented in Sec. I C 3 for both equibiaxial and uniaxial forces and are shown in Suppl. Fig. 14.

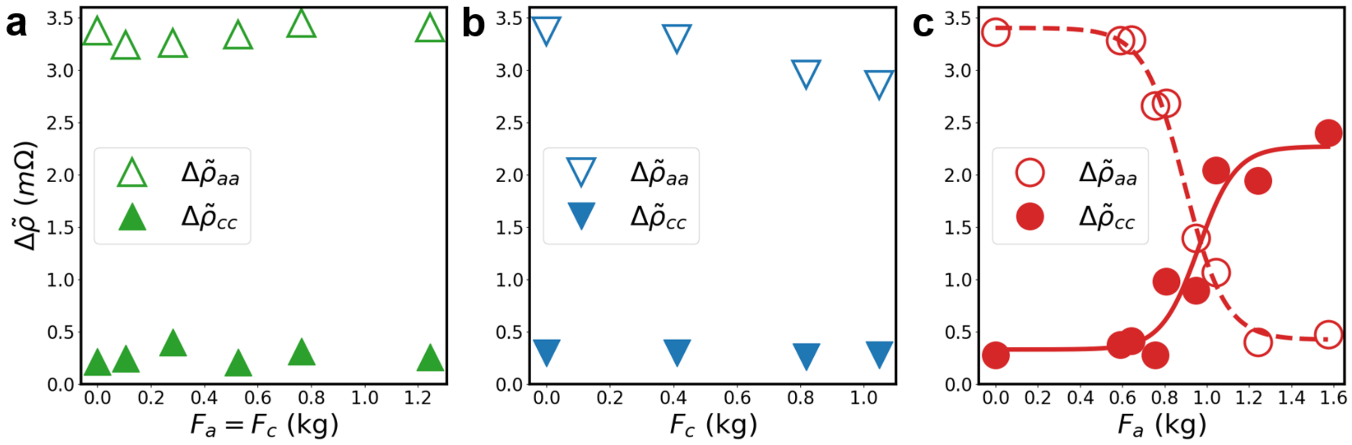

Supplementary Figure 14. **Resistivity jumps obtained for equibiaxial and uniaxial forces in Sample 2.** Resistivity jumps $\Delta\tilde{\rho}_{aa}$ and $\Delta\tilde{\rho}_{cc}$ for (a) equibiaxial forces, (b) uniaxial forces along $\vec{a}$ and (c) uniaxial forces along $\vec{c}$ extracted from the data presented in Fig. 13, as a function of applied forces. The curves representing $\Delta\tilde{\rho}_{aa}$ and $\Delta\tilde{\rho}_{cc}$ in (c) have been fitted with sigmoid functions (dashed and solid lines respectively).

For equibiaxial and uniaxial forces along $\vec{c}$, $\Delta\tilde{\rho}_{aa}$ and $\Delta\tilde{\rho}_{cc}$ do not present variations (see Suppl. Fig. 14(a)-(b)), but similarly as in Sample 1, dramatic variations are observed when applying forces along $\vec{a}$(Suppl. Fig. 14(c)). The features observed in Sample 2 are comparable to the ones shown in the main article for Sample 1, with a decrease of $\Delta\tilde{\rho}_{aa}$ and a concomitant increase of $\Delta\tilde{\rho}_{cc}$ when F_a increases. The two quantities can be fitted with sigmoid and inverse sigmoid curves, crossing at $F_a \sim 0.95$kg. The saturation of both quantities is obtained for $F_a > 1.3$kg, and the saturation value of $\Delta\tilde{\rho}_{cc}$ is again $\sim 65\%$ of $\Delta\tilde{\rho}_{aa}$ value in the pristine state, like in Sample 1.

The variations of T_c were also analyzed quantitatively with the method presented in Sec. I C 4, and are presented in Fig 15.

Like in sample 1, T_c presents a linear decrease as a function of applied forces when $-F_c$ and F_a increase up to $F_a = 0.9$kg, *i.e.* the crossing point of $\Delta\tilde{\rho}_{aa}$ and $\Delta\tilde{\rho}_{cc}$ when F_a is increased (as shown in Fig. 13c). Like in Sample 1, this continuous increase of $-F_c$ and F_a should be correlated to a continuous increase of a/c ratio. Above $F_a = 0.9$kg, T_c displays a linear increase, like also seen in Sample 1. When the sample is deformed in equibiaxial mode though, T_c does not vary (within the ± 1K error bar, as depicted by the green shadowed region in Fig 15).

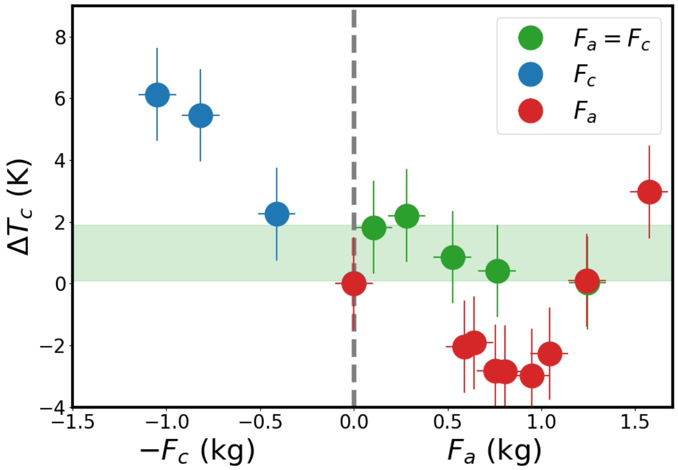

Supplementary Figure 15. **Evolution of T_c in Sample 2 as a function of uniaxial and biaxial forces.** The ΔT_c values are obtained by averaging the T_c values extracted from $\tilde{\rho}_{aa}$ $\tilde{\rho}_{cc}$ and anisotropy with the method presented in Sec. I C 4, and subtracting the T_c obtained in the pristine state ($T_c = 332$K in this sample). Similarly as in the main article, the data points corresponding to forces along $\vec{c}$ (blue dots) are plotted as a function of $-F_c$ for continuity with data obtained with forces along $\vec{a}$ (red dots) plotted as a function of F_a. The data points corresponding to equibiaxial deformation are plotted as a function of F_a, with $F_a = F_c$. The green shaded area represents the constant range of T_c observed in the equibiaxial state (within ± 1K error bar).

The data measured in Sample 2 are thus in perfect agreement with the data measured in Sample 1 and presented in the main text, and show the reproducibility of the phenomenon from one sample to another.

E. Temperature dependence of electron Susceptibility

To find $d\chi/dT$ for the first CDW in the relevant temperature range and for the transfer integrals $t_{\|} \approx 2eV$, $t_{\perp} \approx 0.37eV$ and the Fermi energy $E_F \approx 1.48eV$ in $TbTe_3$ [6], we performed calculations of $\chi\left(T, \vec{Q_0}\right)$ using Eq. 13:

$$\chi\left(T_c, \vec{Q}\right) = \sum_{\alpha,\alpha'} \sum_{k_x,k_y} 16 \frac{n_F\left(E_{\vec{k},\alpha}\right) - n_F\left(E_{\vec{k}+\vec{Q},\alpha'}\right)}{E_{\vec{k}+\vec{Q},\alpha'} - E_{\vec{k},\alpha}} \tag{13}$$

The results are shown in Fig. 16, from which the slope of the temperature-dependent susceptibility can be extracted.

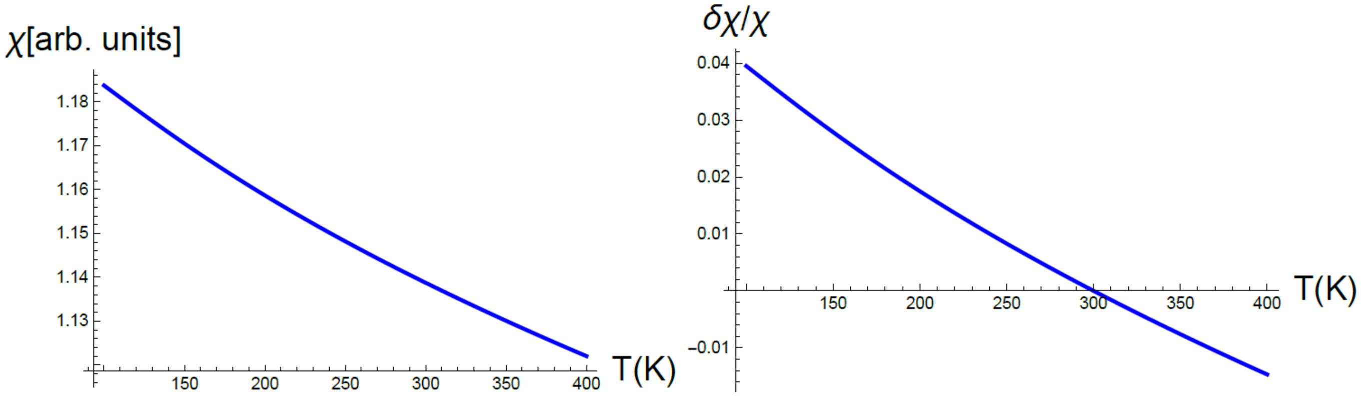

Supplementary Figure 16. . Temperature-dependence of electron susceptibility χ (a) and its difference (b) calculated according to Eq. 13.

II. SUPPLEMENTARY REFERENCES

---

[1] H. C. Montgomery, Method for measuring electrical resistivity of anisotropic materials, Journal of Applied Physics **42**, 2971 (1971), https://doi.org/10.1063/1.1660656.

[2] B. F. Logan, S. O. Rice, and R. F. Wick, Series for computing current flow in a rectangular block, Journal of Applied Physics **42**, 2975 (1971), https://doi.org/10.1063/1.1660657.

[3] N. P. Ong and J. W. Brill, Conductivity anisotropy and transverse magnetoresistance of $nbse_3$, Phys. Rev. B **18**, 5265 (1978).

[4] A. A. Sinchenko, P. Lejay, and P. Monceau, Sliding charge-density wave in two-dimensional rare-earth tellurides, Phys. Rev. B **85**, 241104 (2012).

[5] A. A. Sinchenko, P. D. Grigoriev, P. Lejay, and P. Monceau, Spontaneous breaking of isotropy observed in the electronic transport of rare-earth tritellurides, Phys. Rev. Lett. **112**, 036601 (2014).

[6] V. Brouet, W. L. Yang, X. J. Zhou, Z. Hussain, R. G. Moore, R. He, D. H. Lu, Z. X. Shen, J. Laverock, S. B. Dugdale,

N. Ru, and I. R. Fisher, Angle-resolved photoemission study of the evolution of band structure and charge density wave properties in $r$te$_3$ (r = Y, la, ce, sm, gd, tb, and dy), Phys. Rev. B **77**, 235104 (2008).
